# Multiple Pit Defects in a Foldable Hydrophobic Intraocular Lens

**DOI:** 10.18502/jovr.v15i1.5963

**Published:** 2020-02-02

**Authors:** Arjun Srirampur, Pasyanthi Balijepalli

**Affiliations:** ^1^Anand Eye Institute, Hyderabad, India

Dear Editor,

We are writing to share our observations regarding the article “Multiple pit defects of unknown etiology in a foldable hydrophobic intraocular lens” by Thabit et al.^[1]^ We would like to congratulate the authors for documenting this interesting phenomenon.

The authors mentioned no obvious reason for the formation of pit-like deposits on the anterior surface of the intraocular lens (IOL). We want to disagree with this statement in that IOL pit formation is a well-established phenomenon where opacification of hydrophilic acrylic IOLs occurs after corneal transplantations such as penetrating keratoplasty and Descemet's stripping endothelial keratoplasty (DSEK).^[2]^ Factors such as ocular inflammation and systemic comorbidities that affect ocular metabolism may contribute to the opacification of IOLs.^[3]^ Surgical interventions with the injection of different materials into the anterior chamber such as air or gas seem to increase the risk of IOL opacification, particularly in hydrophilic IOLs. The pits are limited to a more or less circular area of the anterior optical surface of the IOL corresponding to the zone of contact with the instilled air or gas.

**Figure 1:**
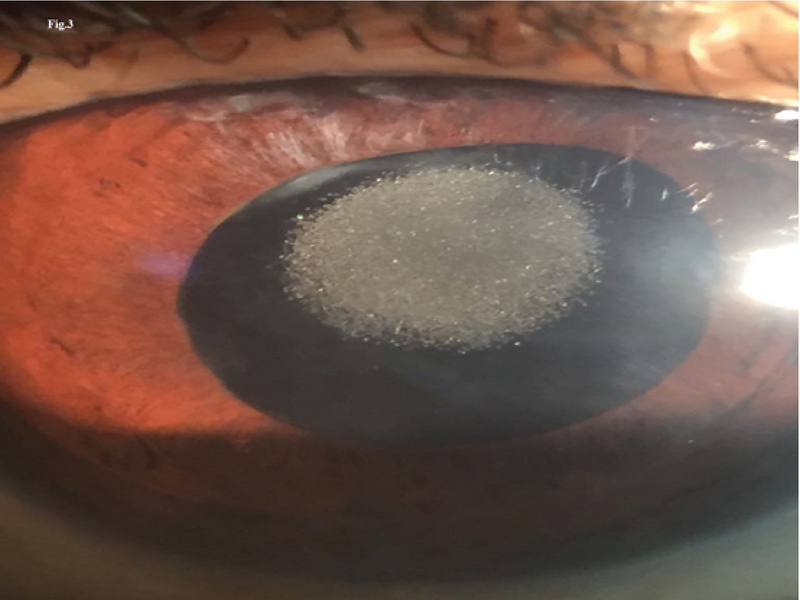
Slit lamp image on diffuse illumination showing the pits confined to the central undilated pupillary area of the intraocular lens.

**Figure 2:**
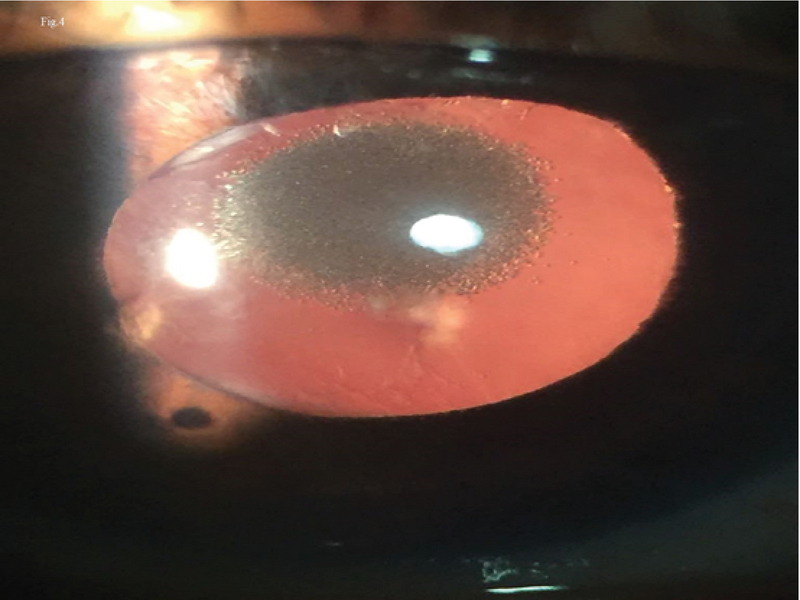
Polychromatic opacification of the pits on the anterior surface of the intraocular lens on retroillumination.

Prolonged breakdown of the blood-aqueous barrier has been suggested as a contributory factor in IOL pit formation. The air in the anterior chamber causes dehydration of the anterior surface of the hydrophilic IOLs, and postoperative inflammation induces a metabolic change in the anterior chamber leading to an increase in aqueous proteins and calcium content that causes subsequent crystallization of the lens.^[4]^ It is our observation that opacification of hydrophilic IOLs develop in eyes that undergo DSEK and receive an air tamponade intraoperatively [Figures 1 and 2].
